# Prognostic values of left ventricular mass index progression in incident peritoneal dialysis patients : a prospective cohort study

**DOI:** 10.1186/s12882-022-02831-6

**Published:** 2022-05-31

**Authors:** Yun Chen, Shuqi Dai, Xiaolin Ge, Da Shang, Qionghong Xie, Chuanming Hao, Tongying Zhu

**Affiliations:** Division of Nephrology, Huashan Hospital, Fudan University, Shanghai, China

**Keywords:** Peritoneal dialysis, Left ventricular hypertrophy, Cardiovascular risks, Survival

## Abstract

**Background:**

Left ventricular hypertrophy (LVH) is common among patients undergoing dialysis. However, the dynamic structural changes of LV are rarely discussed. The study aimed to investigate the prognostic significance of left ventricular mass index (LVMI)-progression in incident peritoneal dialysis (PD) patients, and explore risks factors for LVMI-progression.

**Methods:**

Incident PD patients between February 2008 and July 2018 were recruited. Echocardiography was performed yearly to collect LVMI and evaluate its changes. Participants were divided into three subgroups: group with LVMI-regression, group with LVMI stable and group with LVMI-progression. The end points include all-cause mortality, cardiovascular mortality and cardiovascular events. Cox regression models were performed to identify the associations between LVMI-progression and these endpoints. Multivariate logistic regression was conducted to identify risk factors for LVMI-progression.

**Results:**

A total of 216 PD patients (130 men,60.2%) with a mean age of 54.3 ± 16.8 years were recruited. LVMI-progression was identified in 72 patients (33.3%) after PD initiation. The cohort was followed for a median duration of 65.9 months. Multivariable Cox regression analysis revealed that LVMI-progression was an independent predictor of all-cause mortality (HR, 1.419; 95% CI, 1.016–1.982; *p* = 0.040), cardiovascular mortality (HR, 1.836; 95%CI, 1.084–3.108; *p* = 0.024), and cardiovascular events (HR, 1.494; 95%CI, 1.063–2.099; *p* = 0.021). Multivariable logistic regression showed that hemoglobin, ferritin, blood pressure and fibrinogen were significantly associated with LVMI-progression.

**Conclusion:**

Early LVMI-progression was independently associated with all-cause mortality and cardiovascular outcomes in PD patients. The dynamic monitoring of LVMI might therefore help identify high-risk patients.

**Supplementary Information:**

The online version contains supplementary material available at 10.1186/s12882-022-02831-6.

## Introduction

Cardiovascular mortality in dialysis patients is 10 to 20 times higher than in the general population [1]. Given the far greater cardiovascular risks among dialysis patients, it is important to assess the role of traditional and nontraditional risk factors for cardiovascular disease. Alterations in left ventricular (LV) mass and structure are common in patients with chronic kidney disease (CKD) [2]. Among patients treated by hemodialysis or peritoneal dialysis (PD), it is reported that the prevalence of left ventricular hypertrophy (LVH) is approximately 75% [3].

Echocardiogram is one of the most common imaging tests to evaluate the LV geometry. And high left ventricular mass index (LVMI) is widely used as a quantitative tool to assess LVH [4]. Previous reports have shown that LVH in CKD contributes to high cardiovascular mortality [5–7], and increased LVMI predicts higher mortality in PD patients [8, 9]. LVMI thus has been widely accepted as an indicator of prognosis in dialysis patients. Although many studies have investigated the results of a single echocardiogram in PD patients, very few studies reviewed serial echocardiograms dynamically. As early as in 1992 [10], Hüting J, et al. have found that LVH could be changeable during the long-term CAPD treatment. However, the structural change of LV over time and its impact on PD patients are rarely discussed.

In the present study, we regularly followed up the LVMI of PD patients through echocardiogram. The study aimed to investigate the prognostic significance of LVMI-progression in patients on PD, and explore risks factors for LVMI-progression.

## Materials and methods

### Population and study design

It was designed as a prospective observational study. From February 2008 to July 2018, adult incident PD patients from Huashan Hospital, Fudan University, China were recruited. Patients were excluded if they were transferred from hemodialysis or failed renal transplantation. Patients exhibited an ongoing infection, neoplasia, or unstable cardiovascular disease with a life expectancy of less than 6 months were also excluded. Participants were followed from enrollment until death, a switch to hemodialysis (HD), renal transplantation, a transfer, or March 2020. The study was approved by the Ethics Committee of Huashan Hospital, Fudan University and all participants provided written informed consent.

### LVMI acquirement

In each patient, standard transthoracic echocardiogram was performed at PD initiation and followed up yearly. All measurement data were read and confirmed by professional cardiac sonographer according to the recommendations of the American Society of Echocardiography (ASE) [11]. Left ventricular end-diastolic diameter (LVDd), interventricular septum thickness (IVST), posterior wall thickness (PWT) during diastole, left ventricular ejection fraction (LVEF) and E/A ratio were evaluated and recorded. LVMI was calculated using the following equations [4]:

$$LVM\:=\:0.8\:\times\:(1.04\:\times\:({\lbrack(LVDd)\:+\:(PWT)\:+\:(IVST)\rbrack}^3\:-\:\lbrack LVDd\rbrack^3))\:+\:0.6\;g\;\lbrack g\rbrack$$

$$LVMI\:=\:LVM/(body\;surface\;area)\;\lbrack g/m^2\rbrack$$

### Definition of LVMI changes and the grouping method

LVMIs in the first 2 years after PD initiation were collected. ∆LVMI was calculated to define LVMI changes (LVMI_0_ = the baseline LVMI, LVMI_2_ = LVMI in the second year, ∆LVMI = LVMI_2_-LVMI_0_). Participants were divided into 3 subgroups according to ∆LVMI:

- Group 1: participants with LVMI-regression: ∆LVMI ≤ -5%LVMI_0_;
- Group 2: participants with LVMI stable: -5%LVMI_0_ < ∆LVMI < 5%LVMI_0_;
- Group 3: participants with LVMI-progression: ∆LVMI ≥ 5%LVMI_0_.

### Clinical and laboratory data

Demographic characteristics including sex, age, history of diabetes, smoking status and medication status (use of statins, anti-hypertensive drugs and renin–angiotensin–aldosterone system [RAAS] antagonists) were obtained at the time of enrollment. Height, weight, BSA, body mass index (BMI), blood pressure, laboratory parameters and dialysis-related parameters were also recorded at baseline and collected semiannually. The laboratory data include hemoglobin, albumin, iron metabolism parameters, calcium-phosphate metabolism parameters, lipid metabolism parameters, plasma fibrinogen (FIB), N-terminal pro brain natriuretic peptide (NT-proBNP) and high-sensitivity C-reactive protein (hsCRP). The PD-related data include urinary output, total Kt/V, residual Kt/V, total clearance of creatinine (Ccr), residual Ccr, normalized protein catabolic rate (nPCR), glucose concentration in dialysate and volume of dialysate. Dialysis adequacy was evaluated using Baxter PD Adequest 2.0 software (Baxter Healthcare Corporation, Deerfield, IL, USA). The average values of all these indexes in the first two years were calculated.

### End points

The primary endpoint of the study was all-cause mortality. The secondary endpoints were cardiovascular events (CVEs) and cardiovascular mortality. CVEs were defined when patients exhibited myocardial infarction, acute coronary syndrome, non-hemorrhagic or hemorrhagic stroke, heart failure, arterial embolization, diabetic foot or non-traumatic amputation. Mortality caused by CVEs was defined as cardiovascular death.

### Statistical analysis

Statistical analyses were carried out with SPSS 22.0 (SPSS, Inc., Chicago, IL, USA). Numeric variables were presented as mean values ± standard deviation or as median with quartile ranges. Categorical variables were presented as percentages. One‐way analysis of variance (ANOVA), and two‐way ANOVA analysis were performed in continuous variables to make comparisons while Chi-square test was performed in categorical variables as appropriate. Univariate and multivariate Cox regression models were used to explore risk factors for all-cause mortality, CVEs and cardiovascular mortality and to clarify the associations between LVMI changes and these endpoints. Binary logistic regressions were conducted to identify the risk factors for LVMI-progression.

All statistical tests were 2-sided and a p value < 0.05 was considered statistically significant.

## Results

### Characteristics of study population

The study population included 216 patients (130 men; mean age of 54.3 ± 16.8 years) with a mean BMI of 22.4 ± 3.3 kg/m^2^. LVMI-regression was identified in 113 patients (52.3%) while 72 patients (33.3%) were detected to have LVMI-progression. Clinical data changes in the first 2 years after PD initiation were displayed in Table 1. And Table 2 showed the average values of all these clinical indexes among 3 groups. Compared to patients with LVMI-regression, patients with LVMI-progression had higher blood pressure, NT-proBNP, hsCRP and FIB but lower level of hemoglobin, ferritin and iron saturation. Besides, HDL and ApoA1 were lower in the group with LVMI-progression. No difference was observed in medication status and dialysis-related variables among three groups.

**Table 1:** Clinical characteristics of participants in the first 2 years after PD initiation

	Baseline	1^st^ year	2^nd^ year
Age	54.3 ± 16.8	/	/
Male, n(%)	130(60.2%)	/	/
CAPD,n(%)	202(93.5%)	/	/
BMI (kg/m2)	22.4 ± 3.3	23.3 ± 3.6^*^	23.6 ± 3.8
SBP (mmHg)	140(130,155)	130(117,145)	131(120,141)
DBP (mmHg)	80(73,90)	80(70,90)	80(72,90)
MAP	100.7(93.3,110.0)	96.7(89.7,106.7)^*^	98.0(90.0,106.7)^*^
LVDd (cm)	5.0(4.6,5.4)	4.9(4.5,5.3)^*^	4.9(4.4, 5.2)^*^
PWT (cm)	1.1(0.9,1.2)	1.0(0.9,1.1)^*^	1.0(0.9,1.2)^*^
IVS (cm)	1.1(1.0,1.3)	1.1(1.0,1.3)	1.1(1.0,1.3)
LVMI (g/m^2^)	128.9(107.7, 154.9)	115.9(98.1,136.6)^*^	116.6(93.3,139.6)^*^
LVEF (%)	66.0(62.0,71.0)	67.0(64.0,71.0)	67.0(63.0,72.0)
E/A ratio	0.79(0.63,1.00)	0.73(0.61,0.90)	0.71(0.59,0.87)
Dialysis-related variables			
nPCR (g/kg/24 h)	0.91 ± 0.19	0.95 ± 0.20	0.92 ± 0.22
Urinary output(L/24 h)	1.10(0.63,1,50)	0.80(0.40,1.33)^*^	0.55(0.08,1.10)^*#^
Total Ccr (L/week)	78.4(62.4,96.7)	64.9(52.5,80.2)^*^	54.5(47.5,72.1)^*#^
Residual renal Ccr (L/week)	43.0(27.4,64.2)	30.7(13.6,46.7)^*^	17.1(0.83,38.2)^*^
Total Kt/V	2.04(1.76,2.42)	1.86(1.62,2.14)	1.79(1.58,2.08)^*^
Residual renal Kt/V	0.84(0.50,1.21)	0.63(0.24,0.93)^*^	0.33(0.01, 0.72)^*#^
Volume of dialysate (per 1000 mL/24 h)	5.98(5.51,6.37)	6.21(5.75,7.89)^*^	6.60(6.00,8.70)^*#^
Proportion of 1.5%-glucose dialysate(%)	100(100,100)	70.8(66.7,100)	100(66.7,100)
Biochemical parameters			
Hemoglobin (g/L)	86.7 ± 19.0	106.1 ± 16.7^*^	107.0 ± 16.9^*^
Ferritin (ng/ml)	192.1(86.4,370.5)	212.6(140.7,344.5)	216.9(131.3,305.5)
Serum iron (umol/L)	10.3(7.7,14.1)	12.6(9.9,17.4)^*^	12.8(9.9,16.6)^*^
Iron saturation (%)	27.0(20.0,37.0)	33.0(25.0, 43.0)^*^	32.0(24.0,41.0)^*^
Albumin (g/L)	35.3 ± 5.1	35.9 ± 5.0	36.4 ± 4.8^*^
Calcium (mmol/L)	2.05(1.89, 2.19)	2.18(2.05, 2.34)^*^	2.24(2.09,2.37)^*^
Phosphate (mmol/L)	1.62(1.30,1.97)	1.49(1.25,1.72)^*^	1.49(1.24,1.79)^*^
iPTH (pg/ml)	270.5(158.4,440.0)	261.2(156.5,408.6)	255.5(164.0,389.5)
CHO (mmol/L)	4.13(3.54,5.11)	4.24(3.65,4.93)	4.18(3.61,5.02)
TG (mmol/L)	1.39(1.05,1.98)	1.68(1.15,2.47)^*^	1.72(1.15,2.56)^*^
LDL (mmol/L)	2.33(1.87,2.93)	2.17(1.74,2.66)^*^	2.19(1.72,2.71)^*^
HDL (mmol/L)	0.95(0.81,1.18)	0.91(0.77,1.13)	0.90(0.75,1.12)^*^
ApoA1 (g/L)	0.97(0.84,1.10)	1.00(0.88,1.15)^*^	0.99(0.87,1.17)
ApoB (g/L)	0.65(0.55,0.81)	0.66(0.56,0.78)	0.66(0.57.0.77)
NT-proBNP (per 1000 pg/ml)	1.81(0.83,6.45)	1.31(0.57,3.08)^*^	1.89(0.70,6.41)^#^
hsCRP (mg/L)	2.11(0.47,4.46)	0.86(0.33,2.79)^*^	0.85(0.41,2.67)^*^
FIB (g/L)	3.48(2.95,4.32)	3.80(3.39,4.40)^*^	3.82(3.32,4.45)^*^

*CAPD* continuous ambulatory peritoneal dialysis, *BMI* body mass index, *SBP* systolic blood pressure, *DBP* diastolic blood pressure, *MAP* mean arterial pressure, *nPCR* normalized protein catabolic rate, *Ccr* creatinine clearance, *Kt/V* urea clearance, *CHO* cholesterol, *TG* triglyceride, *LDL* low-density lipoprotein, *HDL* high-density lipoprotein, *NT-proBNP* N-terminal pro brain natriuretic peptide, *hsCRP* high-sensitivity C-reactive protein, *FIB* fibrinogen

^*^p < 0.05 vs. baseline; ^#^*p* < 0.05 vs. 1st year

**Table 2:** Clinical characteristics of participants with different LVMI changes

	Total	LVMI-regression *N* = 113	Stable *N* = 31	LVMI-progression *N* = 72	*P* value
Age	55.3 ± 16.8	54.2 ± 16.8	60.1 ± 16.4	55. 2 ± 17.0	0.785
Male, n(%)	130(60.2%)	65(57.5%)	18(58.1%)	42(64.6%)	0.557
BMI (kg/m2)	23.1 ± 3.5	22.6 ± 3.3	24.2 ± 3.1	23.7 ± 3.6	0.047
SBP (mmHg)	135 ± 14	134 ± 14	135 ± 15	138 ± 15	0.124
DBP (mmHg)	82 ± 8	81 ± 9	80 ± 8	84 ± 10	0.021
MAP	99.6 ± 9.2	98.3 ± 8.8	98.7 ± 8.3	102.1 ± 9.7	0.018
History of smoking	45(20.8%)	23(20.4%)	8(25.8%)	13(20.0%)	0.754
History of diabetes	53(24.5%)	29(25.7%)	4(12.9%)	20(30.8%)	0.253
Use of statins	42(19.4%)	21(18.6%)	6(19.4%)	15(23.1%)	0.931
Use of anti-hypertensive drugs	200(92.6%)	103(91.2%)	28(90.3%)	69(65.8%)	0.436
Use of RAAS antagonists	93(43.1%)	51(45.1%)	11(35.5%)	30(46.2%)	0.130
Dialysis-related variables					
CAPD, n(%)	202(93.5%)	106(93.8%)	27(87.1%)	69(95.8%)	0.251
nPCR (g/kg/24 h)	0.93 ± 0.18	0.94 ± 0.17	0.91 ± 0.15	0.92 ± 0.18	0.647
Urinary output(L/24 h)	0.86(0.50,1.28)	0.83(0.46,1.28)	0.83(0.67, 1.14)	0.92(0.57,1.29)	0.858
Total Ccr (L/week)	67.5(57.1, 81.8)	67.7(57.5,84.0)	68.2(54.6, 88.7)	66.8(57.4,79.5)	0.733
Residual renal Ccr (L/week)	31.3(18.9, 46.5)	32.4(20.1,49.6)	30.5(15.5,52.7)	31.2(18.8,42.9)	0.609
Total Kt/V	1.93(1.72,2.22)	1.94(1.71,2.26)	1.98(1.70,2.16)	1.88(1.73,2.15)	0.539
Residual renal Kt/V	0.61(0.36, 0.88)	0.61(0.35,0.92)	0.61(0.35,0.88)	0.62(0.42,0.87)	0.984
Proportion of 1.5%-glucose dialysate(%)	88.9(69.4,100)	88.9(67.8,100)	80.6(66.7,100)	91.7(75.0,100)	0.770
Volume of dialysate(L/24 h)	6.27(5.82, 7.59)	6.19(5.83,7.53)	6.40(5.92,7.44)	6.30(5.73,7.68)	0.917
Biochemical parameters					
Hemoglobin (g/L)	100.0 ± 13.8	101.8 ± 13.7	99.9 ± 13.7	96.6 ± 13.8	0.055
Ferritin (ng/ml)	233.1(153.8, 327.3)	256.0(163.7,343.2)	207.1(157.0,267.7)	205.8(138.7,307.3)	0.033
Serum iron (umol/L)	12.3(10.1, 15.1)	13.3(10.6,15.3)	12.1(10.4,14.5)	11.6(9.2,14.3)	0.129
Iron saturation (%)	31.7(26.0, 38.0)	32.7(27.3, 39.7)	31.7(27.7, 36.0)	28.3(24.0,34.3)	0.059
Albumin (g/L)	35.8 ± 4.4	36.3 ± 4.7	34.8 ± 4.2	35.3 ± 4.0	0.184
Calcium (mmol/L)	2.15 ± 0.17	2.15 ± 0.18	2.16 ± 0.15	2.14 ± 0.14	0.812
Phosphate (mmol/L)	1.53(1.32, 1.79)	1.56 ± 0.32	1.55 ± 0.36	1.61 ± 0.40	0.513
iPTH (pg/mL)	284.1(186.6, 393.7)	302.3(203.7,423.3)	208.5(156.4,332.3)	283.8(180.8,388.4)	0.139
CHO (mmol/L)	4.40 ± 0.91	4.46 ± 0.99	4.22 ± 0.80	4.40 ± 0.83	0.439
TG (mmol/L)	1.62(1.25, 2.28)	1.64(1.23, 2.57)	1.54(1.32, 2.33)	1.64(1.22, 2.29)	0.994
LDL (mmol/L)	2.29(1.92, 2.71)	2.25(1.88,2.73)	2.29(1.74,2.59)	2.32(2.08,2.70)	0.311
HDL (mmol/L)	0.94(0.80, 1.12)	0.97(0.84,1.15)	0.84(0.76,1.03)	0.90(0.77,1.09)	0.036
ApoA1 (g/L)	0.99(0.89, 1.12)	1.01(0.94, 1.15)	0.93(0.86, 1.09)	0.96(0.86, 1.06)	0.009
ApoB (g/L)	0.69 ± 0.15	0.69 ± 0.16	0.68 ± 0.14	0.70 ± 0.14	0.822
NT-proBNP (per 1000 pg/ml)	2.26(0.93, 7.47)	1.95(0.80,7.58)	2.28(1.46,5.23)	2.50(1.15,8.93)	0.428
hsCRP (mg/L)	1.65(0.79, 4.12)	1.33(0.61,3.73)	2.11(1.12,4.40)	1.96(1.12,5.40)	0.032
FIB (g/L)	3.72(3.26, 4.31)	3.64(3.13,4.13)	3.85(3.34,4.36)	3.88(3.45, 4.59)	0.014

The duration of the observational period was 65.9 (31.9–87.4) months for all patients. Overall, 53 patients experienced CVEs, 56 patients died with 22 of them died from CVEs. More all-cause mortality, cardiovascular morbidity and mortality occurred in the group with LVMI-progression. During the follow-up, 2 patients transferred to other centers, 17 switched to HD and 15 received renal transplantation.

### Association of LVMI changes with all-cause mortality and cardiovascular outcomes

In the group with LVMI-regression, a total of 23 (20.4%) patients experienced CVEs; 20 (17.7%) patients died and 8 (7.1%) died from CVEs. Compared to the LVMI-regression group, more all-cause mortality (28,38.9%; *p* = 0.020) and cardiovascular events (22, 30.6%; *p* = 0.037) occurred in the group with LVMI-progression.

In univariable Cox regression models, LVMI-progression was a risk factor for both all-cause mortality and cardiovascular events while not for cardiovascular mortality (Table 3). In multivariable Cox regression analysis after adjustment, LVMI-progression turned out to be an independent predictor for all-cause mortality (HR, 1.419; 95% CI, 1.016–1.982; *p* = 0.040), cardiovascular events (HR, 1.494; 95%CI, 1.063–2.099; *p* = 0.021) and cardiovascular mortality (HR, 1.836; 95%CI, 1.084–3.108; *p* = 0.024) (Table 4). In addition, a negative correlation was observed between LVMI and LVEF (Supplementary Fig. 1). The correlation coefficient was -0.287 with *p* < 0.001, verifying the adverse role of LVH progression in cardiac function.

**Table 3:** Univariable cox regression analysis for possible predictors of all-cause mortality, cardiovascular events and cardiovascular mortality

	**All-cause mortality**			**Cardiovascular events**			**Cardiovascular mortality**		
	**HR**	**95.0% CI**	**p**	**HR**	**95.0% CI**	**p**	**HR**	**95%CI**	**p**
Age	1.054	1.032–1.076	< 0.001	1.037	1.017–1.057	< 0.001	1.036	1.006–1.066	0.017
Sex	0.792	0.445–1.412	0.430	0.546	0.300–0.914	0.048	0.657	0.268–1.612	0.359
History of diabetes	2.547	1.451–4.470	0.001	2.684	1.556–4.629	< 0.001	2.650	1.142–6.151	0.023
BMI (kg/m2)	1.135	1.083–1.217	< 0.001	1.119	1.032–1.214	0.006	1.069	0.945–1.209	0.287
MAP	0.998	0.967–1.030	0.904	1.021	0.991–1.052	0.181	1.022	0.976–1.071	0.353
Use of statins	1.263	0.553–2.882	0.579	1.639	0.875–3.069	0.123	1.978	0.773–5.509	0.155
Use of RAAS antagonists	0.577	0.317–1.049	0.072	0.575	0.323–1.024	0.060	0.389	0.143–1.055	0.064
Use of anti-hypertensive drugs	0.683	0.271–1.723	0.419	0.895	0.322–2.489	0.832	2.451	0.103–3.256	0.404
LVMI (g/m^2^)	1.010	1.001–1.020	0.027	1.017	1.008–1.025	< 0.001	1.012	0.999–1.026	0.073
LVMI -progression	1.417	1.049–1.915	0.023	1.359	1.015–1.820	0.039	1.475	0.935–2.326	0.095
Hemoglobin (g/L)	0.990	0.970–1.010	0.319	0.996	0.977–1.016	0.717	1.005	0.974–1.038	0.735
Albumin (g/L)	0.954	0.890–1.023	0.187	1.011	0.940–1.088	0.761	1.046	0.938–1.165	0.418
CHO (mmol/L)	1.028	0.748–1.412	0.866	0.971	0.705–1.337	0.857	1.022	0.637–1.640	0.928
LDL (mmol/L)	1.239	0.820–1.874	0.309	1.187	0.790–1.783	0.408	1.243	0.676–2.286	0.484
TG (mmol/L)	0.883	0.659–1.182	0.403	0.977	0.759–1.257	0.856	0.531	0.273–1.036	0.063
HDL (mmol/L)	1.157	0.438–3.057	0.768	0.354	0.115–1.091	0.071	1.867	0.481–7.244	0.367
ApoA (g/L)	1.262	1.108–1.437	< 0.001	1.127	1.103–1.463	0.001	1.315	1.140–1.517	< 0.001
ApoB (g/L)	2.495	0.411–3.309	0.772	1.953	0.293–12.992	0.489	0.682	0.109–29.868	0.682
hsCRP (mg/L)	1.001	0.967–1.037	0.936	1.018	0.992–1.044	0.178	1.004	0.955–1.055	0.884
nPCR (g/kg/24 h)	0.040	0.006–0.256	0.001	0.107	0.017–0.653	0.016	0.222	0.016–3.123	0.265
Urinary output(L/24 h)	0.542	0.309–0.952	0.033	0.856	0.499–1.467	0.571	0.771	0.325–1.830	0.556
Total Ccr (L/week)	0.997	0.985–1.009	0.646	0.998	0.987–1.010	0.786	1.001	0.984–1.019	0.880
Residual renal Ccr (L/week)	0.997	0.986–1.008	0.575	0.998	0.987–1.008	0.650	0.999	0.989–1.009	0.872
Total Kt/V	1.075	0.931–1.241	0.326	1.081	0.931–1.254	0.305	1.148	0.997–1.323	0.055
Residual renal Kt/V	0.814	0.438–1.511	0.514	0.834	0.460–1.511	0.549	0.995	0.403–2.459	0.992
Proportion of 1.5%-glucose dialysate(%)	1.452	0.263–8.014	0.669	1.057	0.172–6.479	0.952	2.080	0.178–24.236	0.559
Volume of dialysate (per1000mL/24 h)	1.319	1.096–1.586	0.003	1.294	1.075–1.557	0.006	1.193	0.898–1.583	0.223

**Table 4:** Proportional hazard ratios for all-cause mortality, cardiovascular events and cardiovascular mortality for LVMI-progression

	**All-cause mortality**		**Cardiovascular events**		**Cardiovascular mortality**	
	**HR (95%CI)**	***P*-value**	**HR (95%CI)**	***P*-value**	**HR (95%CI)**	***P*-value**
Model 1	1.417(1.049–1.915)	0.023	1.359(1.015–1.820)	0.039	1.475(0.935–2.326)	0.095
Model 2	1.405(1.035–1.905)	0.029	1.0381(1.018–1.059)	0.047	1.438(0.905–2.285)	0.124
Model 3	1.424(1.042–1.947)	0.027	1.391(1.012–1.912)	0.042	1.643(1.020–2.645)	0.042
Model 4	1.419(1.016–1.982)	0.040	1.494(1.063–2.099)	0.021	1.836(1.084–3.108)	0.024

Model 1: without adjustment

Model 2: adjusted for age, gender, BMI

Model 3: adjusted for age, gender, BMI, albumin

Model 4: adjusted for age, gender, BMI, albumin, history of diabetes, use of hypotensive drugs, glucose dialysate concentration, LVMI, ApoA1, FIB and NT-proBNP

### Risk factors for LVMI-progression

To explore risk factors for LVMI-progression, participants with LVMI-regression and LVMI-stable were merged as a new group without LVMI-progression. Age, sex, BMI,use of anti-hypertensive drugs, glucose concentration in dialysate, MAP, hemoglobin, ferritin, iron saturation, ApoA1, NT-proBNP and FIB were added to binary logistic regressions. The multivariate analysis showed that MAP, hemoglobin, ferritin and FIB were independent risk factors for LVMI-progression (Table 5).

**Table 5:** Odds ratios for LVMI-progression in incident PD patients based on logistic regression analysis

	**Univariate**		**Multivariate**	
	**OR (95%CI)**	***P*-value**	**OR (95%CI)**	***P*-value**
Age	1.000 (0.982–1.017)	0.974	-	-
Sex	0.765(0.419–1.397)	0.383	-	-
BMI (kg/m2)	1.085(0.995–1.182)	0.064	-	-
MAP	1.061(1.025–1.099)	0.001	1.074(1.029–1.121)	0.001
Use of anti-hypertensive drugs	3.219(0.710–14.591)	0.129	-	-
Hemoglobin (g/L)	0.974(0.953–0.995)	0.017	0.970(0.942–0.998)	0.037
Ferritin (ng/ml)	0.998(0.995–1.000)	0.071	0.995(0.992–0.999)	0.006
ApoA1(g/L)	0.051(0.006–0.402)	0.005	-	-
hsCRP (mg/L)	1.043(1.003–1.085)	0.036	-	-
Fib (g/L)	1.745(1.241–2.453)	0.001	1.800(1.181–2.745)	0.006
NT-proBNP (per 1000 pg/ml)	1.032(0.989–1.076)	0.147	-	-
Proportion of 1.5%-glucose dialysate(%)	0.852(0.132–5.507)	0.866	-	-

## Discussion

In this study, we followed up the patients’ LVMI changes after PD initiation and identified early LVMI-progression was an independent predictor for all-cause mortality, cardiovascular events, and cardiovascular mortality. We also explored the risk factors related to LVMI-progression. It revealed that MAP, hemoglobin, ferritin and fibrinogen were significantly associated with the process. The findings indicated that dynamic follow-up of LVMI after PD initiation might be useful for the early stratification of patients with a high risk of cardiovascular disease and mortality.

Before our research, several observational studies have confirmed the association between LVMI and high cardiovascular mortality in dialysis patients [5, 12, 13]. However, most of them were cross-sectional and performed in prevalent dialysis patients. Only Zoccali C. et al. have monitored LVMI among hemodialysis (HD) patients and verified that changes in LVMI had an independent prognostic value for cardiovascular events in HD patients [14]. Recently, Tamara's group have found that concentric LVH at PD initiation was independently associated with survival in PD patients [15]. Similarly, they advocated early evaluation of LV geometry at PD initiation. Although the group didn’t evaluate changes in LV geometry over time, they suggested that monitoring of LV geometry over time might provide more accurate predictions regarding patients’ prognosis. Our study to some extent could compensate the limitation of their exploration. To our knowledge, it’s the first study that focuses on the relationship between early-stage LVH changes after PD initiation and the prognosis of patients.

According to the suggestion made by the 2007 ESH/ESC guidelines [16], LVH was defined as LVMI more than 125 g/m^2^ in men and more than 110 g/m^2^ in women. Previous reports showed that at the initiation of maintenance dialysis, 70–80% of patients had evidence of LVH [17–19]. Consistent with the data, 73.6% patients in our study was diagnosed with LVH at baseline. It is noteworthy that more than half of the participants (52.3%) in our study got LVMI reduced after 2-year PD treatment. Like our data, Rebic D et al. have observed a significant reduction in LVMI and a significant improvement of LV function after 1-year PD treatment [20]. It is explainable. LVMI is a modifiable factor. For patients with end-stage renal disease (ESRD), PD offers the advantage of continuous dialysis, which is useful in preventing disequilibrium syndrome such as hypertension, poor extracellular volume control, and anemia. As displayed in Table 1, the median LVMI of all participants was 128 g/m2 at baseline and it decreased to 115.9 g/m2 one year later. Concomitantly, parameters such as hemoglobin, blood pressure, NT-proBNP and CRP were all improved one year after PD initiation.

In the univariate Cox regression analysis, we found that both baseline LVMI and LVMI-progression were risk factors for all-cause mortality and cardiovascular events (Table 3). However, when they were put into the multivariate models together, the predictive role of LVMI was diluted while LVMI-progression was still strongly linked to all these endpoints. Therefore, LVMI-progression was related to mortality and cardiovascular risks independently of LVMI and of a large series of traditional risk factors. Compared to a single cross-sectional LVMI, LVMI-changes might be a more powerful indicator for PD patients’ prognosis.

In PD patients, the most prominent hemodynamic parameters include hypertension, volume overload and anemia [21]. Anemia, together with hypertension, has been reported to be one of the main etiological factors for the development of LVH in dialysis patients [22, 23]. Similarly, our results showed that MAP, hemoglobin and ferritin were independently associated with LVMI-progression in PD patients. Fibrinogen is considered as an established predictor of cardiovascular events [24]. In our data, its adverse role in LVMI-progression was further verified. Volume overload is also thought to be a major factor in the development of LVH [25, 26]. In the present study, the association of NT-proBNP with LVMI-progression could not be demonstrated in the logistic regression models. Firstly, NT-proBNP isn’t the gold standard for volume overload. Secondly, this may be due to the short-term of PD duration. Earlier studies have suggested that patients on short-term CAPD were less volume overload than those on long-term CAPD [27]. We only followed up the first 2-year clinical data after PD initiation. Therefore, the role of volume overload might have been attenuated. In addition, antihypertensive treatment didn’t affect LVH progression in our results. It was reasonable. In our study, over 90% of participants used anti-hypertensive drugs. Interindividual variations of anti-hypertensive therapy might be too small to distinguish statistical differences. Nevertheless, antihypertensive treatment can never be neglected in clinic. Our results showed MAP was an independent risk factor for LVMI-progression, illustrating the importance of blood pressure control.

Several limitations remained in this study. First, the study was single-center based with a relatively small sample size. Further multicenter research with more participants is required to confirm our conclusions. Second, number of events especially the cardiovascular death in our study was small and it may affect the statistical power. Some potential risk factors could be missed. Third, the observational nature of this study didn’t not allow us to examine whether therapeutic intervention on anemia etc. could slow LVMI-progression, improving the survival of PD patients. Further studies were needed to explore the causal relationship between them. Finally, we only collected the clinical data in the first 2 years after PD initiation and didn’t further evaluate changes of LVMI over time. It is reported that long-term CAPD is disadvantageous for volume clearance, so it is associated with higher LVMI compared to short-term CAPD [28, 29]. Although a long-time monitoring of left ventricular structure might provide more accurate predictions for patients’ prognosis, our results showed that the long-term outcomes of PD patients could be predicted by LVMI-progression at an early point of PD initiation.

## Conclusion

In conclusion, early LVMI-progression after PD initiation was a predictor for all-cause mortality, cardiovascular events and cardiovascular in PD patients. The dynamic monitoring of LVMI after PD initiation might therefore help identify patients with a high risk of cardiovascular disease and mortality. In addition, the study found that hypertension, anemia, iron deficiency and high level of fibrinogen were independent risk factors for LVMI-progression. These factors could be the possible point for interventions. Further investigations are needed to clarify whether early treatment for factors such as anemia could improve outcomes in PD patients.

## Supplementary Information

**Additional file 1: Supplementary  Figure 1. **Relationship between LVEF and LVMI.

## Data Availability

All data generated or analysed during this study are included in this published article.

## Abbreviations

ASE: Society of Echocardiography
ANOVA: Analysis of variance
BMI: Body mass index
CAPD: Continuous ambulatory peritoneal dialysis
CKD: Chronic kidney disease
CVEs: Cardiovascular events
Ccr: Creatinine clearance
CHO: Cholesterol
DBP: Diastolic blood pressure
ESRD: End-stage renal disease
FGF-23: Fibroblast growth factor-23
FIB: Fibrinogen
HDL: High-density lipoprotein
HD: Hemodialysis
hsCRP: High-sensitivity C-reactive protein
IVST: Interventricular septum thickness
Kt/V: Urea clearance
LV: Left ventricular
LVH: Left ventricular hypertrophy
LVDd: Left ventricular end-diastolic diameter
LVEF: Left ventricular ejection fraction
LVMI: Left ventricular mass index
LDL: Low-density lipoprotein
MAP: Mean arterial pressure
NT-proBNP: N-terminal pro brain natriuretic peptide
nPCR: Normalized protein catabolic rate
PD: Peritoneal dialysis
PWT: Posterior wall thickness
RAAS: Renin-angiotensin-aldosterone system
SBP: Systolic blood pressure
TG: Triglyceride
