# Supplementary figures and images for: Prognostic values of left ventricular mass index progression in incident peritoneal dialysis patients : a prospective cohort study
Source: BMC Nephrol. 2022 May 31;23:200. doi: 10.1186/s12882-022-02831-6 (PMC9158358; doi:10.1186/s12882-022-02831-6)

**Supplementary figure 1:** Relationship between LVEF and LVMI.


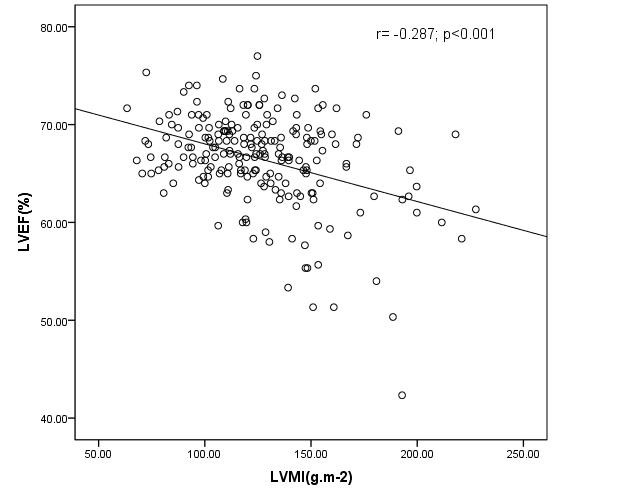

Supplement: Supplementary file 1 — Additional file 1: Supplementary Figure 1. Relationship between LVEF and LVMI. [file 12882_2022_2831_MOESM1_ESM.docx]
